# Participatory design and qualitative evaluation of a decision guide for workplace human immunodeficiency virus self‐disclosure: The importance of a socio‐ecological perspective

**DOI:** 10.1111/hex.13252

**Published:** 2021-05-04

**Authors:** Gayle Restall, Francis Diaz, Patrick Faucher, Kerstin Roger

**Affiliations:** ^1^ Rady Faculty of Health Sciences University of Manitoba Winnipeg MB Canada; ^2^ George & Faye Yee Centre for Healthcare Innovation Winnipeg MB Canada

**Keywords:** community participation, decision support techniques, disclosure, human immunodeficiency virus, risks and benefits, workplace

## Abstract

**Background:**

Disclosure of human immunodeficiency virus (HIV)‐positive status in a workplace can be a complex social decision for a person living with HIV.

**Objective:**

To design a Decision Guide to support people living with HIV in assessing contexts, risks and benefits of workplace disclosure in choosing whether or not, or to what extent, to disclose. In this report, we review the participatory design of a Decision Guide prototype and focus on its evaluation.

**Methods:**

We began with stakeholder input through an environmental scan and community consultation that informed the development of an online Decision Guide prototype. To evaluate the comprehensiveness, acceptability and usability of the prototype, we used qualitative methodology involving individual interviews and the think‐aloud technique. Interviews were transcribed and analysed qualitatively.

**Results:**

Fourteen people, including people living with HIV and service providers, participated. We identified benefits of the Decision Guide related to comprehensiveness, acceptability and usability. Additional interview themes focused on disclosure concerns, mitigating risks associated with disclosure and additional considerations for the Decision Guide.

**Conclusions:**

The Decision Guide was perceived to be acceptable, comprehensive and useful. The findings endorse the application of a socio‐ecological perspective when designing decision support aids for complex social decisions.

**Patient or public contribution:**

People with lived experience of HIV were involved in the prototype design phases as research team members. They, along with community leaders and service providers, also participated in a community forum and were key informants for the evaluation of the Workplace Disclosure Decision Guide prototype.

## 1 BACKGROUND

People living with human immunodeficiency virus (HIV) can live long term in good health interspersed with periods of disability.^1^ Episodes of poor health and disability can increase over time as people experience the cumulative effects of the health challenges of ageing with HIV.^2^ Stigma and discrimination are some of the biggest challenges facing people living with HIV,^3^ making disclosure of HIV‐positive status in community environments such as workplaces a high‐stake decision.^4^ In this paper, we describe the development of a decision support aid to assist people living with HIV to make decisions about whether or not, and the level to which, to disclose their HIV‐positive status in the workplace.

The decision of whether to disclose one's HIV status is complex and may be driven by social pressures to disclose, individual health and social needs and, simultaneously, the desire to protect personal privacy and prevent potential stigmatization and discrimination. Fear of stigmatization can have ripple effects related to unwanted third‐party disclosure with negative consequences in other environments.^5^ The disadvantages of disclosure in workplaces may be compounded for people experiencing intersecting forms of stigmatization (eg men who have sex with men, racialized people, immigrants and refugees) and people who may be economically vulnerable.^6^ Alternatively, many people who have partially or fully disclosed their HIV status in the workplace have reported that they benefited through receiving workplace accommodations, support from co‐workers and decreased stress.^7^ Disclosure can have broader social benefits by providing opportunities for role modelling, education and stigma reduction.^8^ People living with HIV have a right to make decisions about disclosure. A decision aid has the potential for assisting people to make informed decision.

Decision aids have been developed to assist people receiving care to make complex medical intervention decisions for which there is no clear advantage to a particular decisional option. Medical decision aids engage people in decision making by explicating the specific decision to be made, providing information about the options and outcomes, and clarifying values.^9^ In a systematic review, Stacey et al^10^ concluded that decision aids for treatment and screening decisions improve people's active role in decision making, knowledge of risks and benefits, perceptions of being informed and perceived clarity about their values in relation to the decision, while reducing feelings of decisional conflict. We applied the principles and processes of decision aid development to design a Decision Guide for the complex social decision of disclosure decision making in the workplace.

### 1.1 Guiding frameworks

We combined two frameworks to design the Workplace Disclosure Decision Guide: the disclosure process model (DPM)^11^, ^12^ and the Ottawa Decision Support Framework (ODSF).^13^ The DPM proposes that a disclosure decision by a person living with HIV is determined by antecedent goals, the disclosure event, mediating processes and outcomes (personal and social), and a feedback loop that influences subsequent disclosure decisions.^12^ Attention needs to be paid to the mediating contextual (personal and social) factors that will influence the decision and outcomes. In this regard, we recognize the intersecting socially constructed identities (eg race, ethnicity, gender, sexual orientation, ability) that contribute differently to each individual's personal and social circumstances and shape each decision to disclose in unique and multiple ways. Intersectionality^14^ as applied here highlights the importance of analysing and understanding how individuals considering disclosure in the workplace need to examine the risks associated with their HIV status, as well as additional socially stigmatized identities.

We also applied basic elements of the ODSF that combine several decision‐making theories.^15^ The framework provides guidance for decision aid development that meets the person's decision needs including resolving decisional conflict, clarifying the decision and the person's values associated with the decision and creating a good outcome for the person based on relevant information and the person's values.^13^

## 2 OBJECTIVE AND METHODS

The overall objective of the project was to develop a Decision Guide to support people living with HIV to assess contexts, risks and benefits of workplace disclosure in choosing whether or not, or to what extent, to disclose. We begin by describing the process of creating the Decision Guide prototype. We then describe the methods and results of the prototype evaluation.

### 2.1 Prototype design

**FIGURE 1:**
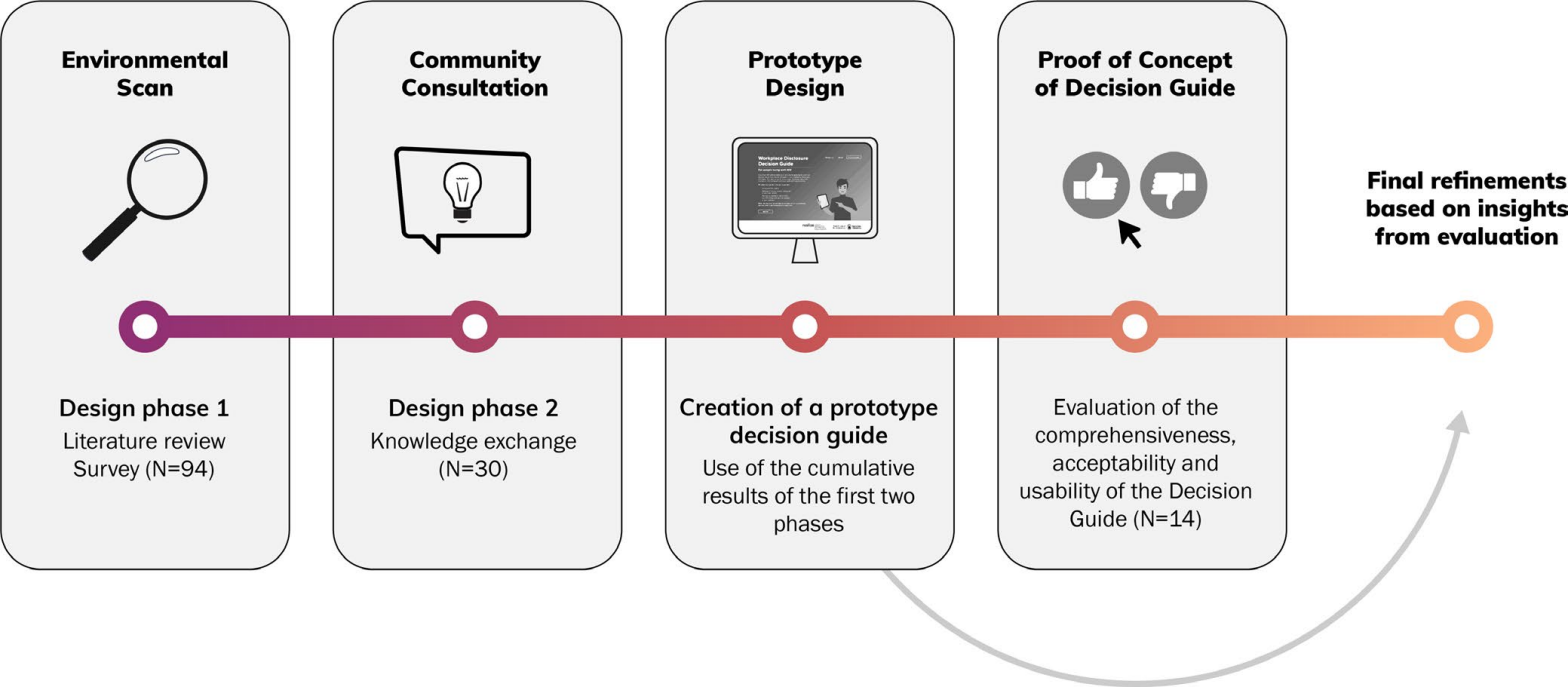
Workplace disclosure decision guide design process

The design process is summarized in Figure 1. We initiated the project by engaging a community‐based research team consisting of researchers, service providers and people living with HIV to complete an environmental scan of needs and supports for decision making for workplace disclosure. The environmental scan included a literature review and cross‐Canada survey of 94 people living with HIV, service providers and other experts in HIV and employment. The results of the environmental scan identified some supports, such as counselling or print information about disclosure, yet a lack of tools to facilitate decision making that are readily available to people living with HIV. Respondents also shared perspectives of important considerations for workplace disclosure decision making. These results are published elsewhere^16^ and not repeated here.

The second phase consisted of a one‐day consultation with 30 participants including community stakeholders from across Canada and the research team. Participants with lived experience were recruited from respondents to the environmental scan survey who agreed to be contacted for follow‐up and recruitment notices sent to HIV‐related organizations. We recruited service providers and policymakers through invitations to people who were part of existing HIV‐related networks. The consultation was approved by the local research ethics board. All participants provided informed consent.

Participants reported that their knowledge of HIV disclosure came from lived experience (n = 16), service provision (n = 13), being a researcher (n = 11) and policymaking (n = 4), with some participants identifying in more than one category. The majority (57%) were female. The average age of the participants was 49 years (SD = 9.4 years). Participants came from a variety of ethnic/racial backgrounds with 16 self‐identifying as Caucasian, White or similar; 6 identifying as Indigenous (First Nation or Métis); seven identifying as African, Black or Latin American; and three participants declined to respond. Some participants identified with more than one group. Two participants reported that they were a landed immigrant or refugee. Of all participants, 13 identified as heterosexual, 16 as gay, lesbian, bisexual or queer; and one declined to respond.

We began a one‐day (6 hour) knowledge exchange process by sharing the results of the environmental scan using deliberative dialogue^17^ and integrated knowledge translation approaches.^18^ We used a World Café^19^ approach to engage participants in small group conversations. Participants responded to three stimulus questions throughout the morning. Question topics focused on what to include in a workplace disclosure decision guide; ways to make it safe and easy for people to access and use a decision guide; and solutions to barriers to using a decision guide. Table hosts collected written notes of the ideas generated. We themed responses to the World Café questions as an interactive process within the large group. After the session, the research team continued to discuss and theme the responses related to the development of a decision guide. Results revealed the components of a Decision Guide that the group deemed most important: education about human and workplace rights, disclosure processes, benefits and risks of disclosure, and maintaining confidentiality. Participants suggested including links to additional resources and ensuring safe access to the Decision Guide for people from diverse cultures, languages, ages, abilities and social circumstances.

To gather more in‐depth feedback from stakeholders, the results from the development phases were transformed into a high‐fidelity interactive (clickable) online prototype Workplace Disclosure Decision Guide using Adobe Xd software (Table 1). The software provided the ability to create relatively quick and low‐cost iterations in response to feedback, allowing stakeholders to more easily influence the direction of the content and its presentation at the critical early stages. Further, creating an interactive prototype allowed stakeholders to provide insights that might not otherwise become apparent.

**TABLE 1:** Workplace disclosure decision guide contents

Page heading	Description
Landing page	Discusses the Decision Guide: for whom and the purpose
Privacy and Confidentiality	Explains that no information is collected during the use of the Decision Guide
How can a Decision Guide be Helpful	Assists individuals to consider the multiple factors before making a disclosure decision
Why is Disclosure a Difficult Decision	Disclosure decisions are complex and consider multiple factors. Disclosure decisions have multiple benefits but also risks associated with them
Your Rights	Directs individuals to links to resources describing their workplace rights regarding disclosure
Your Workplace	Explores the individual's view of safety in the workplace regarding disclosure (eg ‘Do you believe that if you disclose you will be demoted and/or not get a promotion?’)
Extra Support at Work	Explores the individual's need for workplace accommodations due to limitations attributable to HIV
Your Life Situation	Surveys the individual's assessment of his/her/their current life situation (eg health, pressure from family/friends, other reasons to feel excluded)
Your Supports	Explores the supports (eg spouse, partner, family, friends, service providers) available to the individual
Your Values	Explores the individual's assessment of his/her/their own life values (eg openness to sharing personal information)
Your Options	Provides options on to whom, how much and when to disclose, or not to disclose, in the workplace
Making a Disclosure Decision	Allows individuals to weigh the pros and cons of each disclosure decision available to him/her/them
Resources	A list of international and national resources for individuals looking for more information

The prototype was informed by the DPM^11^, ^12^ and ODSF.^13^ We paid attention to a balance of options^20^ by favouring neither disclosure nor non‐disclosure. To take into account that HIV‐related disclosure often occurs on a continuum over time,^21^ we included a range of disclosure options. The development phase of the project highlighted the importance of including a breadth of topics important to disclosure decision making consistent with a socio‐ecologic model,^22^ and we applied this model in the development of the prototype. The socio‐ecological model describes the influence of concentric levels of the environmental context interacting with the individual who is situated at the centre of the model. We applied a version of the model that describes interacting intrapersonal, interpersonal, institutional, community and policy layers.^23^, ^24^ We developed the Decision Guide to provide the user with information related to workplace disclosure decisions prompting consideration of factors at each layer: intrapersonal (eg the person's values), interpersonal (eg relationships with supervisors, co‐workers and people in their social network), institutional (eg organizational culture), community (eg community perspectives about disclosure) and policy (eg legislated rights).

### 2.2 Proof of concept of the workplace disclosure decision guide

The next phase of guide development, and the focus of this report, was to validate the Decision Guide prototype by obtaining the perspectives of key stakeholders about its comprehensiveness, acceptability and usability. We used qualitative methodology involving individual interviews using the think‐aloud technique^25^ in which participants are asked to verbalize their thoughts as they scrolled through an online version of the prototype. We chose the think‐aloud technique and semi‐structured interview questions to obtain detailed perspectives about the content and process of the Decision Guide. This approach facilitated discussion about, and experiences with, workplace disclosure.

#### 2.2.1 Recruitment

We purposefully recruited participants from two groups: (a) people living with HIV and (b) service providers who were clinicians or employment experts (eg employee rights advocates). We sent e‐mail invitations, through the research team's existing networks of individuals and HIV‐related organizations in Canada established through initial phases for the prototype design and previous HIV‐related research. The study was approved by the local university research ethics board. All participants provided informed consent.

#### 2.2.2 Procedures

Participants were interviewed by the second author via web‐based video conferencing software BlueJeans (n = 6, 4 service providers and two persons living with HIV) or in‐person (n = 8; 2 service providers and 6 persons living with HIV). Participants scrolled through each page of the Decision Guide prototype and narrated aloud what they were thinking as they observed each page. The think‐aloud technique encouraged participants to express what worked well, what was missing in the Decision Guide, problems encountered with navigation and concerns such as poor wording or small font. Additional prompts were provided (eg ‘Is there anything that you would add or take out of this page?’). After participants went through the decision guide, a semi‐structured interview guide was used to elicit general feedback on the Decision Guide prototype focusing on comprehensiveness, format, missing information, risks and benefits of using a Decision Guide and ways to increase awareness of the Guide. Interviews lasted between 44 and 100 minutes and were audio‐recorded and transcribed verbatim.

#### 2.2.3 Data analysis

We used NVivo Version 11 to manage data and facilitate analysis. We used both deductive and inductive approaches to qualitative content analysis.^26^ Deductive analysis was used to extract specific participant comments about each page of the Decision Guide related to what participants liked about the page, what was confusing, and what could be improved. We also extracted participants’ impressions of the Decision Guide and any missing information. Inductive thematic analysis was done through line‐by‐line coding of participants’ comments about their own experiences and perspectives related to disclosure decision making. One member of the research team (FD) read all transcripts and conducted line‐by‐line coding. A second member of the research team (GR) reviewed all the transcripts and codes adding additional insights and interpretations to identify primary themes. Results related to the Decision Guide were discussed with additional team members (PF and KR) to finalize actionable feedback that would guide the final iteration of the prototype. Disagreements were resolved through discussion. Changes were incorporated. A summary of findings was sent to participants with a request for comments or feedback.

## 3 RESULTS

Interviews were conducted from April to September 2019. Six service providers and eight community members living with HIV participated. One of the six service providers also identified as a person living with HIV. Average length of time since diagnosis for all participants living with HIV was 18 years (SD = 12.3 years). Average age of all participants was 46 years (SD = 10.1 years). More than half (57%) identified as female. Of the 14 participants, 43% identified as heterosexual and the remainder reported identity as gay, lesbian, queer or bisexual. Nine participants identified as Caucasian or White; three as African, Black or Indigenous; two as Latin American or Caribbean; and one declined to state. Some identified with more than one identity.

We identified four main themes. The first theme was related to the benefits of the Decision Guide. The next two themes were related to the processes of disclosure decision making: workplace disclosure concerns and mitigating risks associated with disclosure. The final theme related to considerations for a final version of the Workplace Disclosure Decision Guide. Description of the themes and illustrative quotes by people living with HIV (P) and service providers (SP) follows.

### 3.1 Theme 1: Benefits of the decision guide

Participants identified several benefits of the Decision Guide. It ‘fuels thoughts’ to support decision making, educates about disclosure rights and reduces stigma associated with HIV.

#### 3.1.1 ‘Fuels thoughts’ to support decision making

Several participants noted that the content of the Decision Guide ‘fuels thoughts’ (P1) and ‘got me thinking’ (P6) about disclosure decisions. One service provider explained that the Decision Guide enables people to acknowledge their thoughts and feelings.

I think it acknowledges the significance of disclosing. It acknowledges people’s feelings around how difficult it can be. It provides them with useful and pragmatic resources … to be able to guide their thinking around it. It doesn’t downplay anything. (SP5)

Moreover, participants noted that the Decision Guide could make it easier for peers to help other community members living with HIV struggling with workplace disclosure. As one community member stated, ‘I will give (it to a peer) and …recommend “go to this … it might be helpful for you”’ (P5). The idea of people using the Decision Guide to consider multiple options for disclosure was reinforced by a service provider who noted that the Decision Guide ‘invites interaction and thought; it doesn't promise a decision but it's useful … to support decision‐making’ (SP5).

#### 3.1.2 Educates people about their rights

There was consensus among people living with HIV and service providers that the Decision Guide was helpful in educating people about their disclosure rights. Several community members living with HIV reported that they can feel pressured to disclose, or not to disclose, their HIV status. One participant noted that the Decision Guide could be helpful for people to learn, ‘what to do in case others come and try and tell you what to do’ (P7). Community members living with HIV asserted that it is imperative for people to know their rights around disclosure. Some participants who were living with HIV for many years did not believe the information about rights was relevant to them because they already knew that information. Thus, the Decision Guide may be more useful for people who are newly diagnosed or do not have ready access to resources about their rights. One participant said:

I guess to help people with HIV and people who need to understand more about HIV… (and) their rights in the workplace, especially for people who are first diagnosed (who) probably think ‘well, you know, do I need to, now that I’m diagnosed with HIV, do I need to tell everyone at work? Do I need to tell my boss?’ … It would be beneficial to them. (P8)

#### 3.1.3 Reduces stigma associated with HIV

Many participants highlighted the stigma surrounding HIV. Some participants believed the Decision Guide could facilitate education and stigma reduction within workplaces and the public. One service provider said:

To be able to share, open up dialogue about HIV/AIDS just generally … say, ‘hey, do you know that this (Decision Guide) is available?’ It just opens the floor up for other discussion… trying to remove some of that stigma. (SP4)

### 3.2 Theme 2: Workplace disclosure concerns

Participants talked about their own experiences and observations about workplace disclosure. Participants living with HIV noted that there are concerns and risks related to disclosing their health status in the workplace. These concerns fell into three categories: lack of confidentiality and protection of privacy; responses of employers and colleagues; and maintaining employment and/or employment benefits.

#### 3.2.1 Lack of confidentiality and protection of privacy

Most participants expressed concern about the lack of confidentiality and protection of their privacy once they disclosed their health status. As one participant summarized, ‘There are no secrets in the workplace’ (P8). Another participant said that disclosure is ‘too big for people not to share it’ (P4). Third‐party disclosure was a concern. A minority believed that their co‐workers ‘disclosed on (their) behalf, thinking that they're going to help you’ (P4). In contrast, most participants believed that third‐party disclosure was related to stigma.

#### 3.2.2 Responses of employers and colleagues

Participants with HIV believed that disclosure could cause co‐workers to treat them differently. Being treated differently could be from co‐workers’ intentions to be helpful or from deep‐rooted stigma. One participant shared how disclosure could cause her co‐workers to treat her ‘with pity…. I want to be given the same challenge, like everybody else’ (P5). In contrast, other participants were concerned about losing the respect of colleagues who ‘would not want to work with me at all’ (P8) if they opted to disclose.

#### 3.2.3 Maintaining employment and/or employment benefits

A few participants shared their concerns regarding obtaining employment or maintaining employment benefits after disclosure. One participant living with HIV shared how they have ‘gone for jobs where there's, you know, a clear question in the application, are you HIV‐positive? And I’ve said to them, I don't think you're allowed to ask that. And then I never got that job. And, and I knew I wasn't going to get that job as soon as I said that’ (P4). The same participant raised concerns about losing their employment benefits after a workplace disclosure. They mentioned that health insurance benefits are ‘the accommodation I would want the most’ and ‘a hard thing to give up if you don't have to’ (P4).

#### 3.2.4 Intersectionality and disclosure decisions

In relaying stories of workplace disclosure or non‐disclosure, some participants highlighted the ways intersecting identities can affect disclosure decisions often in the context of the other supports and services that are available to them. Participants spoke of how gender, immigration status and an additional chronic health condition affected their disclosure decisions. A participant described how gender affected his decisions: ‘Because I’m a gay man, I was much more comfortable talking to women managers than men. Maybe it was an internalized bias on my part based on what kind of stigma and discrimination I had gone through earlier on’ (P1). Another participant, who identified as a recent immigrant, spoke of the lack of culturally appropriate services for immigrants to Canada. One participant, who identified as Indigenous, spoke of accessing Indigenous‐specific HIV resources. The same participant stated feeling more comfortable disclosing other chronic health conditions than disclosing HIV due to the stigma associated with HIV.

### 3.3 Theme 3: Mitigating risks associated with disclosure

Participants shared their perspectives on ways to combat risks associated with intentional or unintentional disclosure of their health status in the workplace. These strategies grouped into four categories: making intentional disclosure decisions, taking assertive action to safeguard privacy, having a caring social circle and having strong and enforced legislation and workplace policies.

#### 3.3.1 Making intentional disclosure decisions

Participants were unanimous about the need to make intentional decisions about whether, when, how much and to whom to disclose in the workplace. A service provider said that some individuals with HIV want to disclose, ‘to as few people as possible; who's the one person that actually needs to know’ (SP6). A participant who had disclosed in the workplace mentioned the importance of timing, stating that they recommend disclosing after the probationary period since, ‘Before that, you're always skating on thin ice’ (P1). Another participant said they recommended not disclosing ‘until it's going to help you’ (P4).

#### 3.3.2 Taking assertive action

Participants acknowledged that confidentiality is not guaranteed when disclosing to an employer. One service provider said, ‘While it is the law that your employer must maintain confidentiality about your health status, that's not always what happens’ (SP5). Some participants with lived experience took pre‐emptive action to educate themselves about their rights prior to disclosure. One participant said, ‘that's why I went through those additional steps (of knowing my rights) so that I would have the legal hammer’ (P1).

Other participants described confronting colleagues who made third‐party health disclosures stating, ‘I went and actually talked to the chain of people. There was no maliciousness … all I wanted to explain to them was this is just something that is private’ (P4).

#### 3.3.3 Having a caring social circle

Having a caring social circle that is not overly protective was highly valued by the participants. One participant ‘started (an) HIV peer support group’ (P1), after disclosing to a colleague. Participants noted that they received support from people with another stigmatized health condition such as hepatitis C. Although participants acknowledged the benefit of having a caring social circle, one participant cautioned against the social circle being overly protective, saying, ‘I don't want to be treated differently, that is why I don't disclose. Not differently in terms of negative, but differently in trying to be too careful’ (P5).

#### 3.3.4 Having strong and enforced legislation and workplace policies against discrimination

Participants acknowledged that the risks of discrimination are still present, despite current legislation in place to protect discrimination in the workplace. One service provider shared that although people who work in health care are bound by legislation to protect personal health information many workplaces do not know about or enforce legislation and policies that protect confidentiality and privacy after a health status disclosure. In addition, pursuing complaints when legislation or policy is not upheld is not always feasible. One participant said:

For a lot of folks living with HIV, they’re fighting stigma all the time and it’s exhausting. So to say, well, to keep your job,…go make a Human Rights complaint (isn’t feasible). (SP6)

Participants voiced the need for stronger and enforced policies against discrimination. One community member shared their experience stating, ‘At my previous workplace there's actually anti‐AIDS graffiti in the bathroom that was never ever cleaned up … and (it's) been there for years’ (P3). Although the graffiti was not directed at an individual, it signalled a culture of HIV stigma and an unsafe workplace to disclose HIV‐positive status.

### 3.4 Theme 4: Considerations for a comprehensive and useful final version of the decision guide

Participants were positive about the comprehensiveness, usefulness and succinctness of the guide. One participant stated ‘I would say it's comprehensive…. I don't feel there's anything missing’ (P5). Another commented ‘I’m not left feeling like I have any unanswered questions’ (P2). The relative brevity of the Decision Guide improved its acceptability for one service provider who noted that ‘[It is] very clean and it's short…. That's wonderful’. (SP5). They found it visually appealing. As one participant noted: ‘In terms of appeal, it works’ (C2).

Service providers, more so than the participants living with HIV, talked about the importance of ensuring a balance between maintaining privacy and confidentiality, and having the ability to save the information generated when working through the guide by, for example, being able to print pages.

Service provider participants wondered about features such as accessibility. They wanted accessibility for people with a disability such as a visual impairment. Participants also suggested that the Decision Guide be accessible across devices (tablets, mobile devices and computers) and in hard copy. As one participant noted, ‘HIV infection covers people from every segment of society. Not everybody has access to a computer and filling it out on paper might be their only option’ (P1). Multiple participants noted that the Decision Guide should be translated into multiple languages including French and Indigenous languages, noting that there would have to be consultation with communities early in the process.

## 4 DISCUSSION

We examined the comprehensiveness, acceptability and usefulness of a Workplace Disclosure Decision Guide that was developed through an iterative process of stakeholder input. Our methodology allowed us to obtain specific feedback about the Guide as well as participant perspectives and experiences with workplace disclosure of HIV‐positive status. The endorsement of participants for the comprehensiveness and acceptability of the Decision Guide highlights the usefulness of decision support aids for this type of complex social decision, a finding that builds on a body of research that has emphasized decision support aids for medical decisions. Our findings also suggest, similar to Durand et al^27^, that the Decision Guide may be most useful to people who have fewer resources, or, as noted in our study, have been recently diagnosed.

One of the features participants found most useful about the Decision Guide was its potential as an educational tool that supported informed decision making. This finding is consistent with Stacey et al’s^10^ systematic review of decision aids that found evidence for increases in people's knowledge after using decision aid tools. Participants in the current study highlighted the comprehensiveness of the information in the Decision Guide. In particular, participants appreciated the information about people's rights. This finding highlights the importance of having breadth of information in a guide for complex social decision making supporting the development of decision guides from a socio‐ecological perspective. Our Workplace Disclosure Decision Guide prompted the user to consider factors at intrapersonal, interpersonal, institutional, community and policy levels. Although values, relationships and some additional ecological factors are often incorporated into diverse decision support aids, policy implications are often neglected or insufficient. For example, Li & Lee applied a socio‐ecological model to explore uncertainty related to health disclosure decisions but focused only on intrapersonal, interpersonal and community levels of influence, neglecting the social policy implications.^28^ In addition, Brohan et al^29^ found that participants in their study wanted more information on legislative issues related to workplace disclosure of a mental health concern.

Previous research has endorsed the empowering effects of people taking control over disclosure decisions.^8^ Participants in the current study endorsed the importance of making intentional decisions about to whom, when and how much to disclose, as well as mitigating the risks of disclosure when possible. Considering the information shared by participants about the on‐going risks of disclosure, such as loss of employment or loss of privacy, careful consideration about managing the disclosure event is warranted. Some empowerment theorists have noted that psychological empowerment is context‐bound and the exertion of power in social decision‐making processes is influenced by both the person and environment.^30^ This understanding supports the usefulness of a decision aid to examine individual factors and contexts to achieve the antecedent goals of disclosure.^11^ Here, too, we acknowledge, as did some participants, the importance of considering intersecting socially constructed identities (eg race, gender, sexual orientation, ability and age) among other factors (eg levels of social support, access to services and economic security) that may make people more or less vulnerable to negative outcomes of the disclosure event. Confidential use of the Decision Guide must consider the complex diverse realities that people live every day.

Studies have found decisional coaching to be useful. For example, Indigenous women in Jull et al’s^31^ study found coaching was important to their engagement in a decision‐making process. Other researchers have suggested that active interventions to support disclosure may help to prevent disclosure regret.^32^, ^33^ Coaching can assist people to enact their disclosure decision within the range of disclosure options.^8^ This can include providing options for protecting one's privacy if the person decides not to disclose or coaching related to the disclosure event if the person decides to disclose. The benefit of formal decision coaching can be explored in future development of the Decision Guide.

The Workplace Disclosure Decision Guide is available free of charge at https://disclosureguide.realizecanada.org. With or without formal decision coaching, an available Decision Guide can be an important resource for service providers and peer support workers, as well as people who do not have expertise in the area of HIV, in providing disclosure decision‐making support to people living with HIV.

The attention we have paid to disclosure decision making does not negate the responsibility of workplaces to create psychologically safer spaces for people living with an invisible disability to disclose their health status.^34^, ^35^ Likewise, policy‐level initiatives to reduce stigmas, repeal stigmatizing and discriminatory laws and processes, and promote workers’ rights are warranted. The Workplace Disclosure Decision Guide can facilitate discussions about these topics in the workplace, adding to the Guide's utility.

### 4.1 Limitations and future directions

We recruited participants from diverse backgrounds and social identities providing a rich source of information for the study. Although the sample size was consistent with sizes for feasibility studies, we may not have identified a full range of perspectives. Increasing accessibility of the Decision Guide to the diversity of people making disclosure decisions while acknowledging the importance of the impact of language, culture and intersecting identities is an opportunity for the next phase of the Decision Guide development. Future research should evaluate the outcomes of the use of the Workplace Disclosure Decision Guide and its applicability for use by people with other episodic health conditions. We acknowledge the prototype's limitations in accommodating accessibility features, which are typically standard in fully functioning websites. Many accessibility barriers have since been addressed within the software and incorporated into the final online version.

## 5 CONCLUSIONS

The iterative approach through which we developed the Workplace Disclosure Decision Guide resulted in a prototype that was perceived as comprehensive, acceptable and useful by participants in this study. Disclosure of HIV‐positive status in the workplace can be a complex decision with risks and benefits. The findings from this study endorse the application of a socio‐ecological perspective when developing decision guides so that people can make high‐quality personal decisions in the contexts in which they live and work.

## CONFLICT OF INTEREST

All authors declare that they have no conflicts of interest with regard to this research.

## Data Availability

The data that support the findings of this study may be available in a condensed or combined form that will not identify participants. The data are not publicly available or are restricted due to privacy or ethical restrictions.
